# Gene Determinants for Mercury Bioremediation as Revealed by Draft Genome Sequence Analysis of *Stenotrophomonas* sp. Strain MA5

**DOI:** 10.1128/MRA.00130-19

**Published:** 2019-04-25

**Authors:** Ashish Pathak, Meenakshi Agarwal, Rajesh Singh Rathore, Ashvini Chauhan

**Affiliations:** a School of the Environment, Florida A&M University, Tallahassee, Florida, USA; University of Arizona

## Abstract

A soilborne *Stenotrophomonas* sp. strain (MA5) that is resistant to mercury was isolated. A draft genome sequence-based analysis revealed a suite of gene determinants to resist mercury and other heavy metals, multidrug efflux, stress response, and membrane transport, and these provide cues to a suite of mechanisms that underpin cellular survival in contaminated soil.

## ANNOUNCEMENT

The Savannah River site (SRS) in South Carolina is a former nuclear legacy site where mercury (Hg) contamination is still pervasive (1). Hg and its microbially methylated form, methylmercury, are toxic environmental contaminants (2); however, microbiota exposed to long-term contamination can recruit genomic mechanisms to resist and detoxify Hg (3). The bacterial *mer* operon, which consists of the *merA* and *merB* gene determinants, drives the detoxification of organometallic or inorganic Hg along with genes that code for regulation (*merR*) and transport (*merT*, *merP*, and/or *merC* and *merF*). Therefore, studies of Hg-resistant bacteria (HgR) can serve as models for understanding the genomic basis of Hg cycling.

Toward this end, several bacterial strains from SRS soils were isolated on LB agar supplemented with Hg (5 µg/ml as HgCl_2_) and incubated at 30°C. The resulting colonies were purified on LB+Hg plates until axenic strains were obtained. Bioremediative mechanisms in strain MA5 are of significant interest to the SRS, because the industrial chloralkali plants located nearby, along with other industrial processes (4), discharged Hg-laden wastes into the surrounding bodies of water, where Hg still poses public health risks. Such environments can be bioremediated using HgR bacteria (5) and fungi (6), a process which results in decontaminated wastewater (7).

To gain a deeper understanding into the genomic underpinings of Hg cycling, a single colony of strain MA5 was picked from a LB+Hg plate and inoculated into liquid LB medium and grown at 30°C in a shaker. After overnight growth, DNA was extracted with Qiagen’s DNeasy PowerLyzer kit and sequenced with an Illumina HiSeq 2000 instrument (8). Default settings of the bioinformatics pipelines were used, unless specified otherwise. Genome *de novo* assembly was performed with CLC Genomics Workbench (v11.0.1; Qiagen, Aarhus, Denmark), and sequences were trimmed with a quality threshold of Q20 and a requirement of 50 bases after trimming. Approximately 8.5 million paired reads (with an average length of 118 bases) were employed for assembly. The nonscaffolded assembly generated 264 contigs, with an *N*_50_ value of 64,365 bases, and a total size of 4,513,544 bases, with an average coverage of 200×.

Rapid Annotations using Subsystems Technology (RAST)-based annotation (9) revealed 3,921 coding sequences and a G+C content of 66.2%. MA5 was taxonomically related to *Stenotrophomonas* spp. with One Codex analysis (10). Annotation resulted in the binning of approximately 48% of the strain’s genome squence under 1,856 subsystems, with the main gene categories (number of genes) being for carbohydrate metabolism (269); cofactors, vitamins, prosthetic groups, and pigments (229); membrane transport (195); resistance to antibiotics and toxic compounds (130); and stress response (119). Several gene determinants for resistance against heavy metals, including the cobalt-zinc-cadmium efflux system, arsenic detoxification system, and chromate-inducible *chrBACF* operon, along with a plethora of membrane transporters, were also identified, and they potentially enable soil survival of MA5.

Further genome mining also revealed possible resistance mechanisms against Hg, which included the presence of *merA*, which encodes the enzyme mercuric reductase (MerA); the periplasmic Hg^2+^-scavenging protein (MerP); and the inner membrane-spanning proteins (MerT and MerE), which are engaged in the transport of Hg^2+^ to the cytoplasm and its reduction by the activity of the MerA enzyme. Finally, strain MA5 also contained the regulatory MerR and MerD proteins.

### Data availability.

This whole-genome shotgun project has been deposited in DDBJ/EMBL/GenBank under the accession number SDHV00000000. The version described in this paper is version SDHV01000000. The genome sequences obtained from strain MA5 have been submitted to the Sequence Read Archive under the accession number SRR8541833.
